# Passion and resilience in esports: the mediating role of passion in the relationship between weakness under pressure and resilience

**DOI:** 10.3389/fpsyg.2026.1819430

**Published:** 2026-05-07

**Authors:** Nahit Ozdayi, Eda Ozkan, Alp Kaan Kilci

**Affiliations:** 1 Department of Coaching Education, Faculty of Sports Sciences, Balikesir University, Balikesir, Türkiye; 2 Department of Physical Education and Sports, Institute of Health Sciences, Balikesir University, Balikesir, Türkiye; 3 Department of Sports Management, Faculty of Sports Sciences, Balikesir University, Balikesir, Türkiye; 4 Directorate of Sports Sciences Application and Research Center, Balikesir University, Balikesir, Türkiye

**Keywords:** e-sports, passion, pressure, resilience, weakness under pressure

## Abstract

**Objective:**

This study aimed to examine the relationships among weakness under pressure, sport passion, and exhibiting resilience in amateur and professional esports players in Türkiye, and to determine whether sport passion mediates the relationship between weakness under pressure and exhibiting resilience.

**Method:**

The present study was conducted with 508 male volunteers aged 18 years and over from various provinces of Türkiye. Data were collected through face-to-face and online settings using a personal information form, the Sport Passion Scale, and the Sports Team Resilience Characteristics Inventory. In the present study, the Cronbach's alpha coefficients of the scales were 0.84 and 0.90, respectively. Normality was assessed based on the ±2 skewness and kurtosis criterion. The data were analyzed using descriptive statistics, independent samples *t*-test, Pearson product-moment correlation, simple linear regression, and mediation analysis using Hayes' PROCESS Macro.

**Results:**

Esports players who had received formal esports training reported significantly higher levels of sport passion and exhibiting resilience. Exhibiting resilience also increased with age and years of esports involvement. Moreover, sport passion was positively and moderately associated with exhibiting resilience and emerged as a significant predictor of exhibiting resilience. Mediation analysis further showed that sport passion partially mediated the relationship between weakness under pressure and exhibiting resilience.

**Conclusion:**

The findings suggest that sport passion plays an important role in resilience-related processes among esports players. Players with higher levels of sport passion appear to demonstrate stronger capacities for exhibiting resilience in the face of competitive pressure and challenges. In this regard, sport passion may be considered a meaningful psychological resource for supporting exhibiting resilience and psychological well-being in esports settings. Therefore, fostering balanced and constructive forms of passion should be considered in the design of psychosocial interventions aimed at strengthening resilience-related capacities in esports players.

## 1 Introduction

The rapidly growing digital gaming ecosystem on a global scale carries e-sports to an important position in terms of economic volume, number of players and viewers. It is predicted that the number of people playing digital games worldwide will reach 3.02 billion in 2029 (Yalçiner et al., 2022). The fact that there are approximately 3,300 licensed e-sports players in Turkey as of 2017 shows that e-sports has been institutionalized as an organized sports field (Aslan and Çoknaz, 2021). Individuals interested in e-sports at a professional or semi-professional level; With intense training schedules, competitive pressure, performance expectations, and online-specific stressors, it is important to examine the psychological characteristics of e-sports players and their relationship with team functioning to understand performance and psychological well-being.

In the psychological literature, passion is defined as long-term interest and investment in an activity that an individual is interested in Vallerand et al. (2003). The Dualistic Passion Model (DMP), proposed by Vallerand and colleagues and based on Self-Determination Theory, conceptualizes passion as a strong inclination toward an activity that individuals find meaningful and enjoyable, leading them to devote a significant amount of time and energy to that activity (Vallerand et al., 2003; Vallerand, 2008, 2010, 2015). Through repeated engagement, such activities can gradually become an integral part of the individual's identity and life. According to the model, passion can manifest in two ways: harmonious passion (HP) and obsessive passion (OP). Harmonious passion reflects an autonomous internalization of the activity and enables individuals to engage in it voluntarily and in a balanced manner with other aspects of life, which typically results in positive experiences. In contrast, obsessive passion represents a more controlled form of internalization, where individuals feel compelled to participate in the activity, which can lead to conflict, emotional tension and negative psychological consequences (Vallerand, 2016). However, the measurement tool used in this study conceptualizes passion as a unidimensional construct reflecting general sports passion (Sigmundsson et al., 2020). Consequently, this research focuses on the level of general sports passion rather than distinguishing between harmonious and obsessive passion, and interpretations regarding passion should be evaluated within this broader conceptual framework.

The concept of resilience generally refers to flexibility and the capacity for recovery. In other words, resilience is defined as the ability of individuals to maintain their functioning by adapting to stressors (Luthar et al., 2000; Masten and Wright, 2010). Team resilience is the group-level counterpart of this concept. In addition to the ability to act as a team, it is the capacity to continue working together in undesirable situations, such as crises or failures, whilst maintaining performance and acting in a harmonious and effective manner. However, in some cases, intense stress and high performance expectations can lead to a decline in performance, despite the resilience capacity possessed by individuals or teams. However, in some cases, intense stress and high performance expectations can cause individuals or teams to experience a decline in performance despite their resilience capacity. Such situations are addressed in the literature as “weakness under pressure” or “performance decline” and are explained by the concept of “choking under pressure”. “Choking under pressure” is a metaphorical expression used to describe the emergence of low performance despite individual effort and situational demands for superior performance (Baumeister, 1984). Pressure can be defined as any factor or combination of factors that increases the importance of performing well in a given situation. Choking refers to a decline in performance under pressure. These terms are most commonly used in the context of competition.

Sports is an important competitive platform where individual talents and intra-team cooperation come together (Türkmen and Ökmen, 2023). However, talent and technical skills alone may not be sufficient for the success desired by the parties. At this point, psychosocial elements such as passion and team resistance are thought to significantly affect success.

This study is based on the assumption that a passion for sport may play a critical psychological role in how esports players respond to performance pressure. In competitive esports environments, players are frequently exposed to intense psychological demands, which can lead to underperformance under pressure. A passion for the activity may influence how players interpret these pressure situations and how they cope with them. Accordingly, passion for sport may function as a psychological mechanism that shapes the relationship between under-pressure vulnerability and team resilience by influencing motivation, commitment and perseverance in challenging situations. In this context, the aim of this study was to examine the mediating role of passion for sport in the relationship between under-pressure vulnerability and team resilience among esports players in Turkey. Furthermore, the study investigated whether levels of passion and resilience differed according to selected demographic and esports-related variables. By focusing on the psychological mechanisms underlying competitive pressure in esports environments, the study aims to contribute to the emerging literature on psychological resilience in digital sports contexts.

For this purpose, the following hypotheses have been developed to test the theoretical mechanism between variables:

*H1: There is a significant relationship between weakness under pressure, exhibiting resilience and sport passion*.

**Figure 1:**
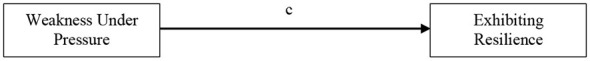
Total effects of weakness under pressure on exhibiting resilience.

*H2: Weakness under pressure significantly predicts exhibiting resilience competence (**Figure 1**)*.

**Figure 2:**
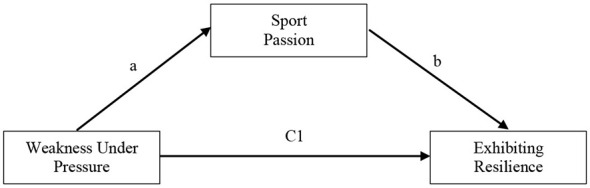
The mediation model proposes that weakness under pressure indirectly predicts exhitibiting resilience through sport passion.

*H3: Sport passion mediates the relationship between weakness under pressure and exhibiting resilience (**Figure 2**)*.

## 2 Materials and methods

### 2.1 . Participants

The study universe consists of players residing in different cities of Turkey and interested in e-sports games. The research sample was determined by using convenience sampling and snowball sampling methods together. As a result, a total of 528 e-sports players were reached in physical and online environments. As a result of the removal of missing data and extreme values from the sample, 508 participants were included in the statistical analysis. When determining the sample size, Sekaran (2003) states that a sample size of 384 is sufficient for studies involving a population of 1 million or more. Accordingly, it was concluded that the sample of 508 participants included in the study was sufficient for the research. All of the participants are male actors and have different age groups and education levels. In addition, some of the participants have an e-sports license and consist of individuals who have received training in this field.

### 2.2 Data collection tools

In the study, demographic information form, Sports Passion Scale and Sports Team Resistance Characteristics Inventory were used as data collection tools.

Passion in Sports Scale: The Passion in Sports Scale, developed by Sigmundsson et al. (2020) and adapted to Turkish by Özdayi et al. (2021), was used to evaluate the level of passion for the game and success. The scale consists of 8 items graded in the five-point Likert type and has no inverse item and sub-dimension. In the original Turkish adaptation study, the Cronbach's alpha coefficient of the scale was reported as 0.81; In this research, it was calculated as 0.84.

Team Resistance Characteristics Inventory in Sports: The Team Resilience Characteristics Inventory in Sports, developed by Decroos et al. (2017) and adapted into Turkish by Görgülü et al. (2018), was used to evaluate resistance characteristics at the team level. The scale consists of 20 items and two sub-dimensions: “Showing Resilience Characteristics” and “Weaknesses Shown Under Pressure”. In the original adaptation study, the Cronbach's alpha coefficient of the inventory was reported as 0.93; In this study, the overall alpha value was calculated as 0.90.

### 2.3 Data collection and analysis

Data were collected from participants in person and in various online settings. Participants were reached face-to-face through the Balikesir Metropolitan Municipality Digital Youth Center and various e-sports centers, and data was collected from participants on digital game broadcasting platforms such as Discord servers and Twitch online. The prepared 8–12 min questionnaire form was delivered to the participants and they were asked to fill it out considering their recent game experiences. All participants voluntarily participated in the study and their informed consent was obtained.

The data obtained from the scales within the scope of the research were analyzed using the SPSS package program. The normal distribution of the data set was examined by taking into account the ±2 SkewnessKurtosis criterion (George and Mallery, 2010). In order to determine the relationship between passion level and team resistance in sports, Pearson Product Moment Correlation Analysis was used because the data were normally distributed. Correlation coefficients were high between 0.70 and 1.00; Medium between 0.70 and 0.30; Between 0.30 and 0.00 was considered as a low level of relationship (Büyüköztürk, 2018). Simple Linear Regression analysis was used to predict the team resistance levels of the participants by their passion levels.

This research used the PROCESS macro, developed by Hayes (2022), to analyze indirect effects and their statistical significance via bootstrapping in mediation analysis. According to Hayes's method, if the indirect effect of the independent variable on the dependent variable via the mediator is statistically significant, the mediator's role is confirmed. This is tested using bootstrapping methods to calculate the confidence interval of the indirect effect (Hayes, 2022). The significance level was determined as *p* < 0.05 in all analyses.

## 3 Results

In accordance with the results presented in Table 1, an independent samples t-test was performed to determine whether participants' sport passion levels varied according to esports training status. The analysis indicated that players who had received esports training (*M* = 4.18) had significantly higher sport passion scores than those who had not received esports training (*M* = 3.81; *t* = 5.147, *p* < 0.05).

**Table 1:** Sport passion levels by esports training Status.

Esports training	*N*	*M*	*Ss*	*t*	*p*
Received esports training	122	4.18	0.74	5.147	0.00
Did not receive esports training	386	3.81	0.69		

*p* < 0.05 independent samples test.

As presented in Table 2, an independent samples t-test was conducted to examine whether the sub-dimensions of team resilience differed according to participants' esports training status. The results showed that players who had received esports training scored significantly higher than those who had not received esports training on the Showing Resilience Characteristics sub-dimension (*M* = 3.93, SD = 0.85 vs. *M* = 3.65, SD = 0.72; *t* = 3.557, *p* < 0.05). Similarly, players who had received esports training also reported significantly higher scores on the weaknesses shown under pressure sub-dimension (*M* = 3.39, SD = 1.10 vs. *M* = 3.12, SD = 0.85; *t* = 2.529, *p* < 0.05).

**Table 2:** Comparison of team resilience sub-dimensions by esports training status.

Sub-dimensions	Status of receiving E-sports training	*n*	*M ±Ss*	*t*	*p*
Showing resilience characteristics	Received esports training	122	3.93 ± 0.85	3.557	0.00
	Did not receive esports training	386	3.65 ± 0.72		
Weaknesses shown under pressure	Received esports training	122	3.39 ± 1.10	2.529	0.01
	Did not receive esports training	386	3.12 ± 0.85		

*p* < 0.05 independent samples test.

The results of the multiple linear regression analysis conducted to determine the predictive power of Passion in Sports and Weakness Under Pressure on Exhibiting Resilience are presented in Table 3. The model was found to be statistically significant (*F*_(2, 505) = 162.435_, *p*<*0.001*), explaining approximately 39% of the total variance (*R*^2^ = 0.391) in the dependent variable. When the standardized coefficients (β) are examined, it is observed that Passion in Sports is the strongest predictor of team resilience (β = 0.472, *p* < 0.001), followed by weakness under pressure (β = 0.318, *p* < 0.001). These findings indicate that both independent variables significantly and positively contribute to the resilience characteristics of the participants.

**Table 3:** Regression analysis results for variables predicting resilience.

Predictors	β	*SE*	*p*	*f*	*R*	*R^2^*
Constant		0.158	0.001	162.435	0.626	0.391
Weakness under pressure	0.318	0.029	0.001			
Sport passion	0.472	0.038	0.001			

As presented in Table 4, Pearson correlation analysis was conducted to examine the relationships among the sub-dimensions of team resilience and sport passion levels. The findings revealed that all correlations were positive and statistically significant at the 0.01 level. A moderate positive relationship was found between Showing Resilience Characteristics and Weaknesses Shown Under Pressure (*r* = 0.424, *p* < 0.01). Team Resilience Inventory scores showed a high positive correlation with both Showing Resilience Characteristics (*r* = 0.879, *p* < 0.01) and Weaknesses Shown Under Pressure (*r* = 0.805, *p* < 0.01). In addition, Level of Passion in Sports was positively correlated with Showing Resilience Characteristics (*r* = 0.544, *p* < 0.01), Weaknesses Shown Under Pressure (*r* = 0.225, *p* < 0.01), and Team Resilience Inventory scores overall (*r* = 0.475, *p* < 0.01). These findings indicate that higher levels of sport passion are associated with higher levels of team resilience.

**Table 4:** The relationship between passion level and team resilience sub-dimensions.

Variables	1	2	3	4
1-Showing Resistance Properties				
2-Weaknesses Shown Under Pressure	0.424^**^			
3-Team Resilience Inventory	0.879^**^	0.805^**^		
4-Sport Passion	0.544^**^	0.225^**^	0.475^**^	

^**^p ≤ 0.01.

**Figure 3:**
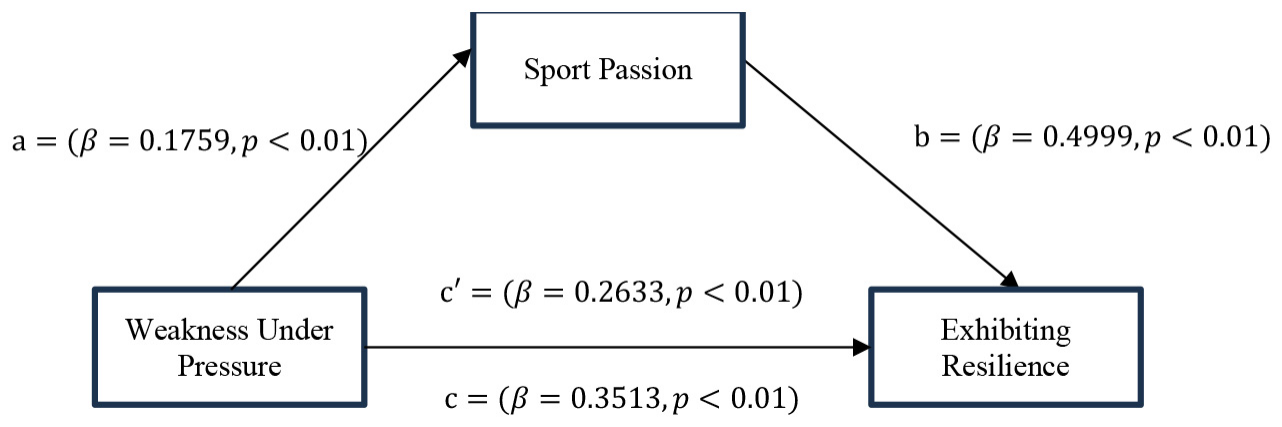
Model results showing sport passion as a mediator between weakness under pressure exhibiting resilience.

According to the PROCESS Macro (Model 4) analysis, which was conducted to determine the mediating role of sports passion in the effect of Weakness Under Pressure on Exhibiting Resilience, all direct paths between the variables are statistically significant. As can be seen in Table 5, the effect of Weakness Under Pressure on Sports Passion (β = 0.1759; *t* = 5.1950; *p* < 0.001) and the effect of Sports Passion on Exhibiting Resilience (β = 0.4999; *t* = 13.2480; *p* < 0.001) are positive and highly significant. The total effect (c) value decreased from 0.3513 to 0.2633 when the mediating variable was included in the model but remained significant (*t* = 8.9268; *p* < 0.001; Table 5 and Figure 3).

**Table 5:** Results of the PROCESS macro: the mediating role of sports passion in the effect of weakness under pressure on endurance.

Pathways	β	*SE*	*t*	*p*	LLCI	ULCI
Weakness under pressure > sport passion (a)	0.1759	0.0339	5.1950	0.000	0.1094	0.2425
Sport passion > exhibiting resilience (b)	0.4999	0.0377	13.2480	0.000	0.4257	0.5740
Weakness under pressure > exhibiting resilience (direct - c')	0.2633	0.0295	8.9268	0.000	0.2054	0.3213
Weakness under pressure > exhibiting resilience (total - c)	0.3513	0.0333	10.5384	0.000	0.2858	0.4168

The results of the bootstrap analysis on a sample of 508 showed that the mediating role of Passion for Sports in the effect of Weakness Under Pressure on Exhibiting Resilience was statistically significant (Indirect Effect = 0.0879; 95% CI [0.0495, 0.1314]). Since the direct effect of weakness under pressure on exhibiting resilience also remained significant, it was concluded that passion for sports partially mediated this relationship (Table 6).

**Table 6:** Bootstrap results on the mediating model of the effect of sports passion on the effect of the process macro on resilience against weakness under pressure.

Paths	Coeff	*SE*	*p*	Boot LLCI	Boot ULCI
Total effects Weakness under pressure > exhibiting resilience	0.3513	0.0333	0.000	0.2858	0.4168
Direct effects Weakness under pressure > exhibiting resilience	0.2633	0.0295	0.000	0.2054	0.3213
**Paths**	**Coeff**	***SE***	**Boot LLCI**		**Boot ULCI**
Indirect effect Weakness under pressure > sport passion > exhibiting resilience	0.0879	0.0207	0.0495		0.1314

## 4 Discussion

The present study was conducted for the purpose of examining the relationship between e-sports players' levels of passion and team resilience, and assessing the predictive role of passion for sport in explaining resilience processes within e-sports environments. The findings indicate that e-sports players generally exhibit moderate to high levels of passion for sport and team resilience. Furthermore, the results of the study demonstrated that receiving esports training is associated with higher levels of both passion and team resilience, and that sports passion is a significant predictor of resilience. Importantly, because passion for sport was assessed using a unidimensional measurement tool in the present study, the findings should be interpreted as reflecting overall sport passion.

It was found that 76.0% of participants (*n* = 386) were actively involved in esports without having received formal training. The analyses showed that players who had received esports training reported significantly higher levels of passion for the sport compared to those who had received no such training. This discrepancy may be attributed to the influence of esports training on the development of commitment and goal-orientation in individuals. The training has been shown to enhance the perceived value and importance attributed to esports participation, suggesting a potential link between training and increased dedication to esports. This heightened dedication may be associated with elevated levels of sport passion.

Recent studies in the field of esports research suggest that formal training and structured development programmes may influence players' levels of motivational commitment and passion (Nielsen and Karhulahti, 2017). Potential explanations for this relationship include the hypothesis that trained players are more inclined to devote greater time and effort to improving their esports performance. In this process, the acquisition of knowledge and skills has been demonstrated to strengthen emotional commitment to the activity and thus contribute to the development of a passion for the sport (Vallerand, 2015; Özer et al., 2015; Sahin, 2017).

Although research focusing on esports is relatively limited, these findings appear to be consistent with broader passion theories in sports psychology (Vallerand et al., 2003; Vallerand, 2008, 2015). From a general theoretical perspective, individuals who devote considerable time, energy, and personal importance to an activity may develop a stronger sense of identification with that activity, which in turn may support the development of sport passion. Concurrently, extant research has demonstrated that specialization, endorsement of autonomy, and identification with an activity contribute significantly to the development of passion (Mageau et al., 2009). In addition, supportive social environments, including coaching support and structured participation, may contribute positively to the development of stronger sport-related motivational attachment. When these findings are considered collectively, it can be posited that regular participation, structured training and psychosocial support processes may contribute to stronger emotional attachment and higher levels of passion within sporting contexts, including esports. For instance, previous studies have shown that training duration, structured coaching, and systematic participation may be positively associated with stronger sport passion and engagement in both sports and esports settings (Huang et al., 2024; Nagorsky and Wiemeyer, 2020; Giakoni-Ramírez et al., 2022; Pedraza-Ramirez et al., 2025).

The findings of this study demonstrated that participants who had undergone esports training exhibited significantly higher scores on the Team Resilience Inventory, particularly in the domains of resilience traits and vulnerability under pressure. This finding may indicate that esports training environments are associated with stronger psychological preparedness and coping resources among players. This finding is largely consistent with research examining psychological resilience and stress management processes in both esports and traditional sports contexts. A comprehensive review of the extant literature on the subject of burnout, resilience and coping strategies among athletes reveals a body of research indicating that resilience functions as a protective psychological resource in the face of stress and competitive demands. Furthermore, this resource can be strengthened through psychological skills training (Poulus et al., 2024; Decroos et al., 2017; Görgülü et al., 2018; Karademir, 2023; Sun, 2022). For instance, Levillain et al. (2024) demonstrated the protective role and mediating effect of resilience regarding burnout among athletes. In addition, research has identified a close association between psychological skills such as stress management and personal efficacy, and both passion and resilience (Ancarani et al., 2024). Similarly, research examining mental resilience among elite esports players suggests that concepts related to psychological resilience may not always directly predict performance outcomes, but remain important for coping with competitive pressure and persevering in challenging environments (Young et al., 2025). When these findings are considered collectively, they suggest that resilience should be understood not merely as an individual trait, but also as a psychological resource that can be developed through structured preparation, training and competitive experience.

The findings of this study have also demonstrated that both a passion for sport and resilience under pressure are significant determinants of resilience. The findings of the study indicated that a fervent enthusiasm for sport emerged as the most significant predictor of resilience levels among esports players. This finding is consistent with previous literature suggesting that a passion for sport may play a significant role in athletes' ability to cope with challenges in difficult situations and maintain psychological resilience. For instance, previous research has shown that passion-related motivational processes may be associated with athletes' resilience and adaptation capacities (Vančáková et al., 2021; Ancarani et al., 2024). The variance in resilience explained in this study (39%) appears generally comparable to previous findings. For example, Mendizabal (2024) reported that perseverance and mental resilience accounted for approximately 32% of athletes' resilience. These findings may indicate that psychological factors such as passion, perseverance and mental strength represent important components of resilience in a sporting context. Furthermore, Zhang and Li (2025) found that the sporting environment positively predicts psychological resilience and that a growth mindset acts as a mediating factor in this relationship.

However, previous studies also show that a passion for sport does not always lead to positive outcomes. Some research suggests that obsessive passion may be associated with negative psychological consequences such as burnout and performance pressure. For example, Demirci and Çepikkurt (2018) reported that athletes with high levels of obsessive passion may be more prone to burnout. Similarly, Beres et al. (2021) found that a tendency to fail under pressure may be positively associated with obsessive passion. Gustafsson et al. (2011) also reported that obsessive passion is positively associated with perceived stress and burnout, whilst harmonious passion tends to be associated with more harmonious psychological outcomes. These findings suggest that the structure and quality of sporting passion may play a significant role in shaping athletes' psychological responses to competitive environments.

At the same time, previous literature suggests that passion may not always be uniformly associated with positive outcomes, and that its relationship with wellbeing, burnout, and pressure-related responses may vary depending on the sporting context and individual experience. However, the present findings indicate an overall positive association between sport passion and resilience in esports players.

The correlation analyses conducted in this study revealed a moderate positive relationship between passion for sport and the sub-dimensions of team resilience. These sub-dimensions include resilience traits and vulnerability under pressure. In addition, a comparable positive correlation was identified between aggregate team resilience ratings and levels of passion. A plethora of studies have previously examined the relationship between passion and resilience in a sporting context. These studies have demonstrated that resilience-related dimensions, such as personal engagement and life adaptation, are significantly associated with passion and may influence psychological performance indicators (Ancarani et al., 2024; França et al., 2020). In addition, it has been documented that athletes who demonstrate higher levels of resilience are able to manage stress and recovery processes more efficiently. This finding suggests that resilience may function as a psychological resource that offers protection in competitive environments (Codonhato et al., 2018). Taken together, these findings support the interpretation that sport passion may act as a meaningful motivational resource associated with resilience processes among esports players.

One of the key findings of this study concerns the mediating role of passion for sport. The results of the PROCESS macro-analysis showed that passion for sport partially mediated the relationship between vulnerability under pressure and resilience. More specifically, higher levels of vulnerability under pressure were associated with higher levels of passion for sport, which in turn were linked to higher levels of resilience. These findings suggest that passion for sport may function as a motivational mechanism linking pressure-related difficulties to resilience processes in esports players. These findings are consistent with several previous studies. For example, previous research has reported that passion-related processes may play mediating or explanatory roles in resilience-related outcomes among athletes (Vančáková et al., 2021; Ancarani et al., 2024; Gu et al., 2022).

In conclusion, the findings of this study suggest that passion for sport, particularly in situations involving competitive pressure, may play a significant role in the resilience processes of esports players. The results indicate that sport passion may serve as an important motivational and psychological resource in coping with pressure and supporting resilience in esports contexts. Therefore, future studies may further examine how different dimensions of passion operate in esports settings using broader measurement approaches.

### 4.1 Limitations

When considering the results of this study, it is important to acknowledge the limitations of the research. The participants of this study were exclusively male esports players, which restricts the generalisability of the results to the broader esports population. It is hypothesized that gender differences may influence how passion, pressure and resilience are experienced in competitive environments. Therefore, future studies involving samples with greater gender diversity could provide a more comprehensive understanding of these psychological processes.

A further limitation pertains to the sampling strategy employed in the study. Participants were selected using convenience and snowball sampling methods, which may lead to sampling bias and reduce the representativeness of the sample. Whilst these approaches are commonly used in esports research due to accessibility constraints, the findings should be interpreted with caution when considering the wider esports community.

The cross-sectional nature of the research design also represents a significant limitation. While the results indicate a clear association between passion for sport, vulnerability under pressure and resilience, the temporal nature of these relationships remains to be definitively established. The employment of longitudinal or experimental research designs could facilitate the elucidation of the causal and developmental dynamics between these variables in future studies.

Furthermore, the study relies on self-report measurement tools. Despite the prevalence of such tools in the field of psychological research, concerns regarding subjective interpretation, response bias, and the influence of social desirability have been raised. It is recommended that future research incorporate additional data sources, such as behavioral observations, performance indicators, or physiological measurements, in order to gain a more comprehensive understanding of the psychological processes of esports players.

Finally, although passion has been theoretically discussed within the framework of Vallerand's Dualistic Passion Model, the measurement tool used in this study operationalized passion as a unidimensional construct reflecting general sports passion. Consequently, this research has not distinguished between harmonious passion and obsessive passion. The utilization of multi-dimensional measurement tools that assess these forms of passion separately could provide deeper insights into the role of passion in resilience and performance processes within the e-sports context.

## 5 Conclusion and recommendations

The findings obtained within the scope of this research reveal that sports passion functions as a partial mediating variable in the relationship between individuals' weakness under pressure and their endurance. It has been determined that passion for sports increases the psychological resilience levels of individuals by alleviating the negative effects of stress experienced under oppressive conditions. In this context, it is stated that athletes with compatible passion can cope with challenging situations more effectively; On the other hand, it is understood that obsessive passion can have limited or negative consequences on endurance.

Based on these findings, it is important for coaches and sports psychologists to design intervention programs that support the development of adaptive passion in athletes. It is considered that psychological skills training, which focuses on stress management, self-awareness and motivational balance, will strengthen both the individual endurance capacities and long-term performance sustainability of athletes. In addition, effective structuring of psychological support units in sports clubs can contribute to maintaining the relationship between passion and weakness under pressure in a balanced and healthy way.

In conclusion, this study shows that passion for sports is not just a source of motivation; it is also an important psychological protective element that supports the development of resilience under pressure. The findings obtained from the study, which reveal that the levels of both passion and team resistance sub-dimensions of players trained in e-sports are high, indicate that structured training processes and professional support mechanisms can strengthen the psychological resources of the players. Training programs are not limited to the transfer of technical and tactical knowledge; Including psychological competencies such as stress management, communication skills, team role clarity and coping strategies can reinforce the positive interaction between passion and resilience (Gu and Day, 2007; Posner and Eiler, 2013; Poulus et al., 2023). In other words, in order to ensure sustainable performance and psychological wellbeing in the context of esports, player passion should be considered as a constructive and protective element.

When evaluated in the application dimension, e-sports clubs, coaches and psychological counselors; In order to support teams in developing high levels of team resilience, they can plan various activities, events and branch-specific training programs to increase the players‘ passion levels for the sport. In this process, it is important that the relationship that players establish with e-sports is shaped in line with the harmonious passion. Especially in the early stages of young players' careers, instead of approaches that focus only on technical performance; It is recommended to support holistic development programs that include psychological resilience and team interaction processes.

## Data Availability

The data supporting the findings of this study are available from the corresponding author upon request.
